# How practice nurses engage with parents during their consultations about the MMR vaccine: a qualitative study

**DOI:** 10.1017/S1463423621000256

**Published:** 2021-05-27

**Authors:** M.C. Hill, D. Salmon, J. Chudleigh, L.M. Aitken

**Affiliations:** 1 School of Health Sciences, City, University of London, Northampton Square, London EC1V 0HB, UK; 2 Menzies Health Institute Queensland, Gold Coast Campus, Griffith University, Queensland 4222, Australia

**Keywords:** practice nurse, factors, influence, strategies, measles–mumps–rubella vaccine, health promotion, immunisation

## Abstract

**Aim:**

We aimed to understand practice nurses’ perceptions about how they engage with parents during consultations concerning the measles, mumps and rubella (MMR) vaccine.

**Background:**

The incidence of measles is increasing globally. Immunisation is recognised as the most significant intervention to influence global health in modern times, although many factors are known to adversely affect immunisation uptake. Practice nurses are a key member of the primary care team responsible for delivering immunisation. However, little is known how practice nurses perceive this role.

**Methods:**

Semi-structured interviews were undertaken with 15 practice nurses in England using a qualitative descriptive approach. Diversity in terms of years of experience and range of geographical practice settings were sought. These interviews were recorded, transcribed verbatim and open-coded using qualitative content analysis to manage, analyse and identify themes.

**Findings:**

Three themes were derived from the data: engaging with parents, the informed practice nurse and dealing with parental concerns: strategies to promote MMR uptake. During their consultations, practice nurses encountered parents who held strong opinions about the MMR vaccine and perceived this to be related to the parents’ socio-demographic background. Practice nurses sought to provide parents with tailored and accurate sources of information to apprise their immunisation decision-making about the MMR vaccine.

## Introduction

Immunisation has been cited as the most significant intervention to influence global health in modern times (World Health Organization, 2020). National immunisation programmes have resulted in a steady decline in child morbidity and mortality (Haider *et al.*, 2019). Vaccines, such as the measles, mumps and rubella (MMR) vaccine, protect against these diseases by conferring immunity (Hakim *et al.*, 2019). However, in order to confer immunity to a significant portion of a population (referred to as herd immunity), the World Health Organization (WHO) recommends that 95% of vaccine eligible people are immunised against vaccine preventable diseases (Haider *et al.*, 2019). Therefore, it is important for health professionals, such as practice nurses, involved in the delivery of national immunisation programmes to strive to achieve herd immunity levels for MMR.

Several factors have been cited as influencing parental immunisation decisions concerning the MMR vaccine. These include socio-demographics such as ethnicity, social class, sources of information (e.g., family, friends, social media), immunisation history, access to immunisation services, weakening the immune system, risk perception of vaccine preventable diseases and information from health care professionals (Forster *et al.*, 2016; 2017; Mixer *et al. et al.*, 2007; Hilton *et al.*, 2007; Austin *et al.*, 2008; Hackett, 2008; Lamden & Gemmell, 2008; Bystrom *et al.*, 2020). Other factors reported to influence parental immunisation decision-making related to fear of vaccination side effects, distrust in the MMR vaccine and the influence of the anti-vaccination lobby reported in the media (Larson *et al.*, 2015).

The incidence of measles has been increasing globally with 9.8 million cases of measles and 142 000 deaths in 2018 (World Health Organization, 2019a). However, by November 2019, case numbers had risen dramatically and had tripled compared with the same period in the previous year (World Health Organization, 2019b).

Data from the European region revealed 82 596 people contracted measles in 2018 (Thornton, 2019). The majority of measles cases were linked to two countries namely: Ukraine (53 218) and France (2,913) (Gallup, 2019). The United Kingdom (UK) has also seen an increasing incidence in measles from 124 cases in 2017, rising to 611 cases in 2018 (Public Health England, 2019). However, the incidence of measles in England and Wales has recently shown a reduction with data for the first quarter in 2020 revealing 507 measles cases compared to 648 cases for the first quarter in 2019 (Public Health England, 2020a).

Practice nurses have been identified as one of the key health care professionals involved in the delivery of national immunisation programmes in the UK (Maconachie & Lewendon, 2004; Joyce & Piterman, 2011). The Chief Nurse for Public Health England has endorsed the significant contribution of practice nurses as leading the delivery of these immunisation programmes (Bennett, 2019). Furthermore, the Royal College of Nursing has affirmed the important public health role of practice nurses in the delivery of national immunisation programmes (Royal College of Nursing, 2018). Therefore, this study addresses the important aim which is to understand practice nurse perceptions about how they engage with parents during consultations concerning the MMR vaccine. This information is particularly relevant and necessary due to the increasing incidence of measles globally. The secondary aim is to ascertain what strategies practice nurses use to promote the MMR vaccine.

## Methods

### Design

In our study, we used a qualitative descriptive approach to explore a phenomena, which was to gain an understanding of the perspective of practice nurses concerning their MMR consultations with parents (Graneheim *et al.*, 2017). Qualitative descriptive studies offer a comprehensive summary of an event, and researchers conducting such studies seek an accurate accounting of events or of participants’ meanings (Sandelowski, 2000). The use of a qualitative descriptive approach allowed us to gather rich descriptions about the phenomenon being explored in an area where there was minimal research. The consolidated criteria for reporting qualitative research (COREQ) checklist was used in the reporting of this study (Tong *et al.*, 2007); see Supplemental Table 1.

### Participants

Convenience sampling was used to recruit participants. A flyer was distributed to practice nurse fora in London and to a national association of general practice nurse educators. All practice nurses who responded to the initial study invitation consented to participate in the study, with none withdrawing their informed consent. The inclusion criteria included practice nurses who were employed to administer the Healthy Child Programme: Pregnancy and the First 5 Years of Life (Department of Health, 2009). The exclusion criteria consisted of: all other registered nurses who were not employed in general practice; not registered on the Nursing and Midwifery Council in the UK or not involved in the administration of the national immunisation programme.

### Data collection

Semi-structured one-to-one interviews were conducted from May to October, 2019. Questions were developed: to ascertain the factors that influence practice nurses in their consultations with parents about the MMR vaccine; the strategies they use to guide these consultations; the information sources used and practice nurses’ education needs concerning the MMR vaccine. See Appendix 1 for the interview questions. Interviews were undertaken by a research assistant either by telephone or at a venue of choice identified by the participant. Interviews lasted between 14 and 44 min and audio-recorded by a research assistant, purposefully employed who did not have a background in the areas of immunisation and public health. This was to remove potential bias and distortion in the study results that may have occurred if the principle author (MH) had been the interviewer. This was due to her involvement in immunisation education, which some participants’ may have been exposed to.

### Data analysis

Interviews were analysed using qualitative content analysis. This form of analysis involves precise reading of textual matter, where relevant parts of the text are coded into analytical categories (Krippendorff, 2019). The use of qualitative content analysis in this study enabled MH to determine how practice nurses engaged with parents during their MMR consultations. The analysis started with identifying certain words or content in the text (i.e., in this case the practice nurse interviews) with the purpose of understanding the contextual use of the words in these interviews (Krippendorff, 2019).

During the coding process, MH defined all codes from the interviews in a coding manual. MH and JC independently coded two transcripts. Following discussion, the coding manual was refined until there was consensus between both authors. MH then coded the remaining 13 transcripts, which were critically reviewed by at least one of the other co-authors (LA or DS). This resulted in a process of discussion amongst all authors. This process continued until there was concordance on the codes, subthemes and themes amongst all authors (MH, LA, JC and DS). This was an iterative process until there was agreement on the final number of themes, which were engaging with parents, the informed practice nurse and dealing with parental concerns: strategies to promote MMR uptake.

### Rigour

Credibility was evidenced through the process of peer debriefing with the co-authors (LA, DS and JC). A characteristic of good qualitative research is for the inquirer to make their positon explicit in their writings. This is the concept of reflexivity (Creswell & Poth, 2018). Reflexivity in research improves transparency in the researcher’s subjective role, which includes conducting research and analysing data, and allows the researcher to apply the necessary changes to ensure the credibility of their findings (Darawsheh, 2014; Dean, 2017). One of these considerations was who would undertake the study’s interviews. In this study, while MH made her position explicit as the lead investigator in the participant information sheet for the study, she confirmed that a research assistant would undertake all interviews.

## Results

Fifteen practice nurses consented to be interviewed; all were female. There was diversity in the academic levels of participants’ nursing qualifications. These ranged from certificate (*n* = 3); diploma (*n* = 3); degree (*n* = 7); postgraduate diploma (*n* = 1) and masters (*n* = 1). Participants described their self-identified ethnic origin as: White British (*n* = 9); White European (*n* = 2); Australian (*n* = 1); British Asian (*n* = 1); South American (*n* = 1) or Caribbean (*n* = 1).

Five participants were employed full time (37.5 h/week) and the remaining 10 were employed part time from 16 to 36 h/week. The length of time these participants were employed as a practice nurse ranged from 8 months to 30 years (Median 17, Mean 15). Thirteen were from London, two were from Derby, England.

### Themes

The principle focus of this study was to ascertain how practice nurses engaged with parents during their consultations concerning the MMR vaccine. Qualitative content analysis yielded three themes: engaging with parents, the informed practice nurse and dealing with parental concerns: strategies to promote MMR uptake.

### Engaging with parents

Practice nurses described encountering parents who held strong opinions about the MMR vaccine, which they perceived as contributing to vaccine hesitancy. In this regard, parents were either refusing the MMR vaccine or conflicted on whether to immunise their children or not. Practice nurses reported that parents refused the MMR vaccine without articulating a reason or were concerned that their child’s immune system was too immature to receive this vaccine.

*I have had situations as well where, a child’s come in for their, let’s say eight-week jabs, and the mum brings up MMR immediately that they don’t want to have it. Obviously I explain that they don’t have it until they’re a year old anyway* (PN 4, 2019)

*We have a few families and, who think that their children’s immune system is too immature at one [year], and so they’ll come back maybe when they’re four or five [years of age]* (PN 8, 2019)

The practice nurse participants’ highlighted the socio-demographics of their practice population and how this influenced parental immunisation decision-making. This related to how different cultures perceived the MMR vaccine, especially those from an Eastern European or Somali background.

*We also have quite a few Eastern Europeans who decide not to give any vaccinations at all, not just with measles, mumps and rubella; any vaccinations* (PN 1, 2019)

*…we do have a Somali population where I work and they tell me that they have a lot of Autistic Spectrum Disorder among the children in their community, and they worry that if they give their own child, when they are still one at this stage, if they give them the MMR vaccine, the child will get the same condition* (PN 3, 2019)

Practice nurses acknowledged parents’ decisions and sought to ensure that parents were in receipt of accurate information concerning the MMR vaccine. Practice nurses displayed understanding about the differing cultural perceptions and dilemmas of their practice populations relating to the MMR vaccine.

### The informed practice nurse

It was important for these practice nurses to have a strong evidence base in order to engage with parents. Practice nurses advised parents about the importance of their children receiving vaccines at the appointed times as delineated in the national immunisation programme, especially if their children were late receiving their vaccines. This was particularly evident in relation to the MMR vaccine. Practice nurses provided contemporary sources of information to assist parents with their immunisation decisions and expertly dealt with questions concerning vaccine content and side effects.

*I always give what’s recommended at the right time, unless the parents, obviously, have forgotten and they arrive late. So, if they arrive late for their 13 months or their preschool boosters, where MMR is one of the vaccinations, I will give it to them. I’d say, ‘It’s better to have it than not to have it* (PN 2, 2019)

*Then, obviously, we need to show them [parents] our immunisation schedule. So, once we show it to them and explain the effect, the side effect, they’re quite happy to go on and take it* (PN 9, 2019)

Although PN recognised the importance herd immunity, they were not always confident that parents understood the definition of herd immunity. Despite this, practice nurses revealed how achievement of herd immunity levels protected those children who could not receive this vaccine, especially when there were local outbreaks of measles and mumps.

*…the only thing I want to say is I think we practice nurses, we all want the uptake to be great, we all want to get the herd immunity* (PN 7, 2019)

*…we have had an outbreak of, of measles and mumps in this area, and we can say, ‘Look, these diseases are coming back. It’s only because we’re getting good herd immunity that will actually protect. ‘We’re also protecting the more vulnerable children; the ones who can’t have it for whatever medical condition that they may have* (PN 10, 2019)

A key part of practice nurses’ consultations involved dealing with parental questions about the MMR vaccine, especially about the gelatine content of one of the two MMR vaccines available in the UK national immunisation programme. Gelatine is a substance derived from the collagen of animals and porcine gelatine (Public Health England, 2020b). In our study, practice nurses advised parents there was an alternative MMR vaccine available without gelatine.

*There may be an issue around the gelatine content with the measles, mumps and rubella because of our patients often a lot of them are Muslim so we explain we have got a measles, mumps and rubella vaccine that has no gelatine in it* (PN 1, 2019)

*But, the other one [MMR vaccine] also uses pork gelatine, and pork gelatine is not accepted by certain communities because of their religious beliefs* (PN 3, 2019)

Practice nurses also endeavoured to reassure parents and confirm that they understood vaccine side effects.

*…once we get their consent, once we give them all the information and make sure that they really thoroughly understand the side effects. A lot of counselling, reassurance* (PN 9, 2019)

Practice nurses advised parents to access recommended sources of information about the MMR vaccine, such as NHS websites and leaflets. Furthermore, they cautioned parents about relying on certain internet sources.

*I try and encourage all parents to use the NHS website…and I also urge a little bit of caution with online fora and looking into the background of any advice that they’re taking from the internet. We always have leaflets available to back things up for the relevant age group or the immunisations* (PN 5, 2019)

*I usually go on the NHS website, print information about MMR. I also direct them to the Public Health [England] and the immunisation site* (PN 12, 2019)

As well as ensuring parents had access to the most contemporary immunisation information, practice nurses were encouraged to avail themselves of immunisation updates by their employers, so that their knowledge was current and evidence-based.

*And then on the NHS web…they do a lot with immunisation. Every immunisation change, they send to us through an email and sometimes there’s a touch of eLearning training as well* (PN 7, 2019)

*…where I work they provide us with, with regular updates. We have like three updates a year, in the classroom, immunisation updates* (PN 13, 2019)

Practice nurses highlighted the importance of having a strong evidence base concerning changes to vaccines in the national immunisation programme. This was to ensure that they were able to address parental questions, as well as directing parents to reputable websites and information sources about the MMR vaccine.

### Dealing with parental concerns: strategies to promote MMR uptake

Practice nurses described that a major concern expressed by parents related to their perceived link between MMR and autism. Parents made an association between MMR and autism, as autism was often diagnosed around the time of the first MMR vaccine.

*And so that’s when you diagnose it [Autism] and that goes hand-in-hand with having an MMR vaccine. So, they just associate the autism with the MMR, don’t they, rather than that’s just when you start to diagnose these things* (PN 8, 2019)

*…they seem to think it [MMR] has some relation to autism, and both of the parents concerned have got older children with autism* (PN 13, 2019)

Practice nurses reported that parents expressed their reservations about the number of vaccines recommended in the national immunisation programme. Consequently, they sought to diffuse these concerns by reassuring parents about the safety of the number of vaccines infants received at any one time and how an infant’s immune system could cope with receiving multiple vaccines.

*…it’s mainly the number of vaccines on the children, they’re very worried about, and we have to reassure them they’re very, very small doses* (PN 3, 2019)

*…some parents just think having three vaccines is too much in one go…we point out that, if their child puts their hand in mud then in their mouth, it’s getting thousands of germs, and things that their immune system is going to have to cope with. And their bodies can easily cope with these multiple vaccines* (PN 15, 2019)

Practice nurses used a number of different strategies to promote the MMR vaccine that included recommending parents have an initial appointment with the practice nurse to discuss vaccines prior to an immunisation appointment. However, practice nurses were keen that parents were not pressurised into making a decision and offered parents the opportunity to return for further appointments prior to making a decision.

*I mean, in my ideal world we’d have…an appointment before the immunisation appointment, where me and parents can sit down and discuss everything and explain what all the vaccines are and why we give them* (PN 4, 2019)

*…I think the most important thing, really, is to try and not get into conflict with people, to leave the door open* (PN 5, 2019)

Practice nurses were aware of the variety of information sources that influenced parents’ immunisation decision-making. These included family, friends and online sources. Practice nurses acknowledged that not all parents’ information sources were credible.

*…maybe they haven’t got access to the internet in the kind of area that I’m working in, and there’s too much relying on word-of-mouth from friends or family* (PN 5, 2019)

*…and often their information doesn’t come from any real scientific basis; it’s usually something that they’ve heard or they’ve read online on a chat group or something* (PN 11, 2019)

Practice nurses noted the influence that measles outbreaks and travel to countries with a high incidence of measles had on parental immunisation decision-making. This led to, in some instances, parents requesting the MMR vaccine prior to when infants would be recommended to have their first MMR vaccine at 12 months of age.

*Sometimes they [parents] hear of an outbreak and they’re quite keen. I think last summer there were a lot of people travelling back to Eastern Europe or they were going off to Israel to visit the areas where there were outbreaks of measles, and they were coming in with their children under a year and wanting them to have the MMR* (PN 11, 2019)

Practice nurses identified how religious leaders influenced some parents MMR decision-making.

*…there was an outbreak of MMR with the Jewish community…and the way we got through to them [parents], we went through the rabbi and the rabbi told everyone to come. So, uptake is now great* (PN 7, 2019)

Practice nurses continued to deal with the legacy of the now retracted Wakefield *et al.* publication in their consultations with parents (Wakefield *et al.*, 1998). Despite the duration of time since this publication and subsequent retraction, parents still continued to express concerns about the alleged link between the MMR vaccine and autism. This made it important for practice nurses to discuss and explain these discredited research findings with parents.

*…again, about autism and about Andrew Wakefield’s research. That still keeps coming back, even though it’s been disputed and thrown out. And it doesn’t seem to matter how often we say, ‘The Autism Society actually recommends that you have it. ‘There’s no proof’… it’s still coming through, even after all these years* (PN 10, 2019)

*The Lancet, published a paper by Dr Andrew Wakefield, and there was a very small cohort, but he was trying to prove or disprove that there is a link between autism, and bowel disease and, the administration of the measles, mumps and rubella vaccination* (PN 14, 2019)

In summary, practice nurses identified a number of strategies to promote the uptake of the MMR vaccine. Their ability to engage with parents was facilitated by their robust evidence base to address parental concerns and provide reassurance about the MMR vaccine.

## Discussion

Practice nurses endeavoured to provide tailored information to assist parents’ immunisation decision-making, especially about the MMR vaccine. They considered how parents’ immunisation decisions were influenced according to their socio-demographic characteristics and by their religious beliefs. Practice nurses worked with religious leaders to provide guidance to members of the community they served. It was important for these practice nurses to have a contemporary evidence base to be able to address these parental concerns and dispel misinformation concerning the MMR vaccine.

In our study, practice nurses were attuned to how parents’ socio-demographic characteristics influenced their immunisation decisions. Practice nurses described using strategies that were tailored to address concerns specific to different ethnic backgrounds. This is consistent with the key recommendations made from a survey-based study of adolescents and parents to increase uptake of adolescent immunisations in the United States (Greenfield *et al.*, 2015). This survey concluded that health care professionals needed to be aware of differing health beliefs amongst ethnic groups to enable them to tailor their consultations to address cultural-specific vaccine concerns (Greenfield *et al.*, 2015). Tailoring consultations to a specific ethnic group to increase immunisation uptake was found to be effective in an intervention study in New Zealand (Turner *et al.*, 2017).

Tailoring involves the provision of information to a specific individual based on characteristics related to the areas of interest that are unique to that person (Kreuter & Skinner, 2000). The purpose of tailoring information is to increase the relevance of the message. Communicating with messages that are specifically tailored to an individual has been found to be more effective than broad ranging messages at changing behaviour (Conway *et al.*, 2017). However, there have been mixed results about the effectiveness of tailored interventions. A randomised trial tested standard care discharge instructions compared to discharge instruction in combination with an information prescription individualised to each patients learning style preference in hypertensive patients in the United States (Koonce *et al.*, 2011). In this trial, there was no significant difference between the groups in hypertension knowledge, although the group that received the tailored intervention reported higher satisfaction scores (Koonce *et al.*, 2011).

In our study, practice nurses identified parents’ frequent use of online sources of information, many of which practice nurses perceived as not credible. This in turn led practice nurses to caution parents on the use of certain online sources of information and guided them to use recommended sources to apprise their MMR decision-making. Furthermore, these practice nurses needed to articulately and sensitively deal with the legacy of the now retracted Wakefield study and diffuse misinformation about this article. In this regard, practice nurses ensured that parents had accurate data about the Wakefield paper, which was guided by their strong and contemporary immunisation evidence base. There is minimal understanding why particular individuals and societies are sensitive to misinformation about health. This has led to health promotion and public health researchers paying attention to the potential of the internet as a tool to spread health-related information (Chew & Eysenbach, 2010). A systematic review to explore the spread of health-related misinformation on social media revealed that there is an increasing trend in published articles on health-related misinformation, with the most commonly associated topics concerning misinformation relating to vaccination (Wang, McKee *et al.*, 2019). Findings from an online survey in Indonesia revealed that the sharing of information on social media without verification was predicated by a number of factors, such as internet experience and belief in the reliability of the information (Khan & Idris, 2019). This survey additionally identified that the perceived self-efficacy of individuals to detect misinformation on social media was predicted by their income and educational level (Khan & Idris, 2019).

All practice nurses in our study ensured they had access to contemporary sources of immunisation information and all reported attending yearly immunisation updates. Furthermore, these practice nurses described availing of other immunisation sources of information to supplement their knowledge to ensure that their clinical practice was evidence-based. Lifelong learning through continuing professional development (CPD) is an essential component to provide health care professionals with the opportunity to keep updated in their clinical practice (Rankin & Armstrong, 2017). It has been contended that CPD is an integral part of both professional and personal development to actively promote critical reflexivity and higher-order thinking in relation to professional practice (Hayes, 2016). In England, The Code contains the professional standards that registered nurses, midwives and nursing associates must adhere to in order to maintain their registration with the Nursing and Midwifery Council (Nursing and Midwifery Council, 2018). One of the four professional standards in The Code is to practise effectively and to do so, registrants must ensure they always practise with the best available evidence and maintain the knowledge and skills required for safe and effective practice (Nursing and Midwifery Council, 2018).

### Implications for practice

Our study illustrates how practice nurses engage with parents to promote the MMR vaccine. The study findings’ emphasises how practice nurses need to take into account different parental socio-demographic characteristics during their MMR consultations. All practice nurses in our study reported attending annual immunisation updates and accessed other recommended immunisation sources of information. A key recommendation for training is to incorporate strategies to enable practice nurses to engage with parents from different socio-demographic groups to tailor their MMR consultations. Many of the practice nurses in our study needed to deal with misinformation. It would be beneficial for annual updates to deal with strategies to counteract misinformation in the media.

### Strengths and limitations

Despite the well-documented role of practice nurses in national immunisation programmes, there is limited description of how practice nurses’ perceive their role during their consultations with parents concerning the MMR vaccine. The sample was self-selected and therefore, this group of practice nurses could be a highly engaged group within their professional group. Although a small number of the participants in our study practiced in locations outside London, further research is needed to ascertain whether similar themes exist across wider geographical areas in the UK. The study is further limited by a lack of a wider advisory group or patient and public involvement and this is recommended for more extensive studies.

## Conclusion

Practice nurses in our study were attuned to the many factors that influenced parental immunisation decision-making about the MMR vaccine, including socio-demographics, online sources of information, family and friends.

They tailored their consultations with parents to take into consideration these factors. In order to mitigate against misinformation, practice nurses signposted parents to recommended NHS websites to inform their immunisation decision-making. Our study has identified the extent to which practice nurses engage with, and promote, the uptake of the MMR vaccine manifested by the strategies they utilised in their practice.

## Supplementary material

For supplementary material accompanying this paper visit http://dx.doi.org/10.1017/S1463423621000256. click here to view supplementary material

## Appendix 1. Practice Nurse 2019 interview questions

- Can you tell me about a typical working week as a practice nurse?
- Can you tell me about the size and population of your general practice?
- What are the challenges in your practice area relating to immunisation?
- How do you communicate to parents concerning the MMR vaccine?
- What are the challenges facing your consultations in relation to the MMR vaccine?
- When a parent attends for the MMR vaccine, tell me what you would say to them?
- How do you deal with parents who are uncertain about vaccinating with the MMR vaccine?
- How informed are parents before coming to see you concerning the MMR vaccine?
- Where do parents get their information concerning the MMR vaccine?
- Where do you recommend parents to get information?
- How do you keep up to date with changes to the national immunisation programme, particularly the MMR vaccine?
- Are you able to avail of opportunities to keep up to date with changes to the national immunisation programme, especially the MMR vaccine?
- What specific information do you need about the MMR vaccine when either attending immunisation updates or accessing online information?
- Has the process and requirements around revalidation influenced these opportunities?
- What is your general practice’s uptake for MMR at 12 months and at school age?

At the end of the interview, elicit the special category data

- How would you describe your own racial or ethnic origin?
- Can you describe your gender?
- What are your formal qualifications?
- In relation to your continuing professional and personal development and immunisation, can you discuss what this is to date?
- Can you tell me how long you have been working as a registered nurse?
- Can you tell me how long you have been working as a practice nurse?
- Are you working as a practice nurse on a full- or part-time capacity and how many hours per week?
